# Design and Fabrication of Microelectrodes for Dielectrophoresis and Electroosmosis in Microsystems for Bio-Applications

**DOI:** 10.3390/mi16020190

**Published:** 2025-02-07

**Authors:** Mengren Wu, Zijian Liu, Yuan Gao

**Affiliations:** 1 Department of Mechanical Engineering, Stanford University, Stanford, CA 94305, USA; 2 Department of Mechanical and Industrial Engineering, University of Illinois Chicago, Chicago, IL 60607, USA; zliu232@uic.edu; 3 Department of Mechanical Engineering, University of Memphis, Memphis, TN 38152, USA

**Keywords:** microfluidics, dielectrophoresis (DEP), electroosmotic flow (EOF), microelectrode design, bio-applications

## Abstract

Microfluidic technology has emerged as a multidisciplinary field, integrating fluid dynamics, electronics, materials science, etc., enabling precise manipulation of small volumes of fluids and particles for various bio-applications. Among the forms of energy integrated into microfluidic systems, electric fields are particularly advantageous for achieving precise control at the microscale. This review focuses on the design and fabrication of microelectrodes that drive electrokinetic phenomena, dielectrophoresis (DEP) and electroosmotic flow (EOF), key techniques for particle and fluid manipulation in microfluidic devices. DEP relies on non-uniform electric fields to manipulate particles based on their dielectric properties, while EOF utilizes uniform electric fields to generate consistent fluid flow across microchannels. Advances in microelectrode fabrication, including photolithography, soft lithography, and emerging non-cleanroom techniques, are discussed. Additionally, the review explores innovative approaches such as rapid prototyping, contactless electrodes, and three-dimensional structures, along with material considerations like conductive polymers and carbon composites. The review discusses the role of microelectrodes in enhancing device functionality, scalability, and reliability. The paper also identifies challenges, including the need for improved fabrication reproducibility and multifunctional integration. Finally, potential future research directions are proposed to further optimize DEP- and EOF-based microsystems for advanced biomedical and diagnostic applications.

## 1. Introduction

George Whitesides, one of the pioneers in the field of microfluidics, defines microfluidics as “the science and technology of systems that process or manipulate small volumes of fluids (10^−9^ to 10^−18^ L) using channels with dimensions ranging from tens to hundreds of micrometers” [1,2,3]. Microfluidic technology has emerged from the convergence of diverse fields, including fluid dynamics, optics, electronics, acoustics, and materials science, among others. This multidisciplinary development has enabled the miniaturization of large bench-top laboratory instruments into portable devices, capable of manipulating not only fluids but also particles [1,4,5,6]. Its ability to reduce sample volumes, accelerate reaction times, and provide cost-effective detection has driven its rapid adoption in biological applications such as drug screening, single-cell analysis, drug delivery, biosensing, and point-of-care diagnostics [7].

These extended functionalities have been advanced through integration with external energy. Electrical energy is particularly advantageous for scaling down and integrating with microfluidic systems. On micro scales, electric fields are becoming increasingly influential, making electrical energy highly effective for manipulating fluids and particles [8]. As a result, it has been extensively applied in microfluidic devices to enable efficient fluid control and particle manipulation. With the electric field, microfluidic systems utilize precisely engineered microchannels and electrodes components to control fluid flow and particle movement at a scale where conventional fluid dynamics differ significantly from macro-scale behavior. At these small scales, the manipulation of fluids and particles is governed primarily by surface forces, electric fields, and capillary action [9,10]. The ability to precisely control these factors allows microfluidic devices to perform tasks such as sorting [11], mixing [12,13], pumping [14], and enrichment [15]. Electrokinetic techniques, including dielectrophoresis and electroosmosis, play a pivotal role in achieving such precise control, making them useful in various microfluidic applications in recent years [16,17,18,19,20].

**Figure 1:**
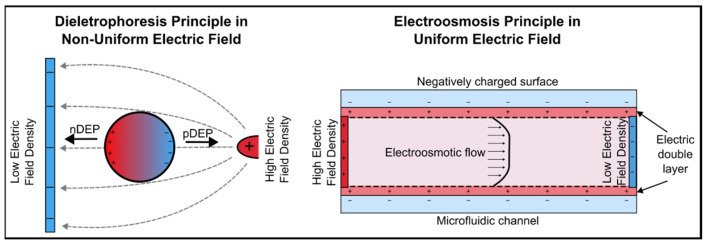
Schematic illustration of dielectrophoresis and electroosmotic flow (EOF) principles.

Dielectrophoresis (DEP) results from the interaction between non-uniform electric fields and dielectric particles [21]. This interaction generates a force that induces particle motion relative to the suspending medium, with the direction and magnitude of the force determined by the dielectric properties of both the particle and the surrounding fluid, as well as the electric field gradient (Figure 1). When particles are attracted to regions of high electric field intensity, the phenomenon is referred to as positive DEP (pDEP). Conversely, when particles are repelled from regions of high electric field intensity and move toward areas of lower field strength, it is known as negative DEP (nDEP). The ability to control particle motion using pDEP and nDEP enables precise manipulation of cells and nanoparticles by exploiting differences in dielectric properties, which have bio-applications in particle and cell sorting [22,23], biomarker concentration [24,25], rotation for imaging [26], and trapping for single cell analysis [27]. Different from DEP’s non-uniform electric field, electroosmosis, another key electrokinetic phenomenon, is characterized by the movement of liquid relative to a charged stationary surface under an applied electric field [28]. This movement results from the interaction of ions in the electrical double layer at the channel wall, leading to bulk liquid flow known as electroosmotic flow (EOF) [29]. Unlike pressure-driven flow (PDF) in microchannels, EOF is plug-like and uniform across the channel, minimizing dispersion and enhancing analyte transport in microfluidic systems [30]. Its controllability makes it suitable for fluid handling and sample injections in micro-total analysis systems (μTAS) [31].

**Figure 2:**
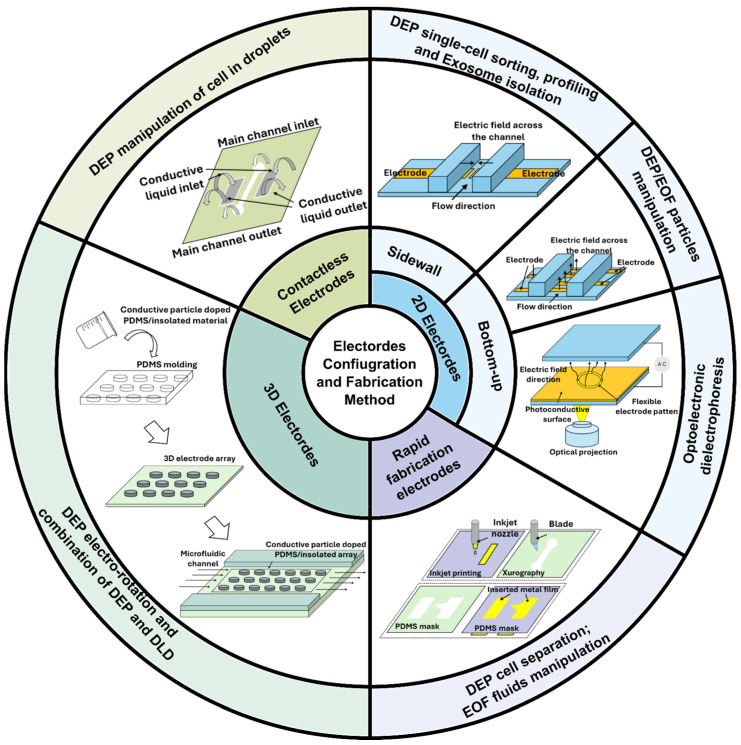
Overview of electrode configurations and fabrication methods in microfluidic devices.

The effectiveness of DEP and EOF in microfluidic systems largely depends on the design and fabrication of microelectrodes, which generate the electric fields for these electrokinetic phenomena. The fabrication process involves precise patterning of microelectrodes onto substrates using techniques such as photolithography [32], thin-film deposition [33], and etching [34]. These processes allow for the creation of microelectrodes with various geometries and materials to optimize field gradients for DEP or achieve uniform electric fields for electroosmotic flow control. For DEP applications, microelectrodes are typically designed to produce non-uniform electric fields that can selectively manipulate particles based on their dielectric properties [35]. Common electrode configurations include interdigitated [36], quadrupole [37], parallel plate [38], coplanar [39], concentric ring [40], asymmetric [41], and 3D microelectrode designs [42], as well as innovative liquid electrodes [43]. Each configuration offers unique advantages, such as enhanced particle capture efficiency, field distribution, or precise control in three-dimensional spaces. The choice of materials, such as gold [44], platinum [45], carbon [46], and silicon [47], further influences the electrode performance due to their conductivity and biocompatibility. In electroosmosis, microelectrodes are used to establish uniform electric fields across microchannels, creating consistent electroosmotic flow. The fabrication techniques ensure smooth electrode surfaces and robust electrical connections to withstand prolonged operation in liquid environments. Advances in fabrication methods, such as micro-electro-mechanical systems (MEMS) technology and soft lithography, have expanded the capabilities of microelectrode integration, enabling more complex and functionalized designs [48,49]. In this review, we will provide a detailed review of recent fabrication techniques, material considerations, and microelectrode designs for optimizing DEP and EOF in microscale systems for bio-applications. Figure 2 shows an overview of this review.

## 2. Microelectrodes for DEP

In this section, we review the fabrication methods for DEP advanced microfluidic chips, focusing specifically on devices designed for biological applications. The discussion is centered on fabrication techniques that enhance device functionality within these systems, with particular emphasis on microelectrode design and channel integration. We limit the scope to actuator-based applications to provide a focused analysis on DEP-based actuation mechanisms for fluid and particle manipulation.

### 2.1. Rapid Fabricated Electrodes

**Figure 3:**
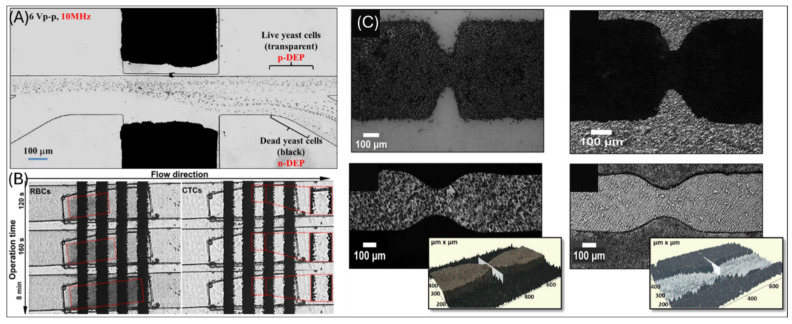
(**A**) Copper-embedded PDMS microfluidic channel for DEP-based separation of live (transparent) and dead (black) yeast cells [50]. (**B**) Inkjet-printed electrodes on xurography-fabricated channels for CTC and RBC separation [51]. (**C**) Sequential fabrication of constriction patterns using inkjet printing, heat transfer, wet etching, and PDMS micro molding [52].

To achieve a low-cost, time-efficient fabrication process for electric microfluidic devices, several non-cleanroom methods have been developed in recent years. These approaches offer alternatives to cleanroom-based fabrication, providing rapid prototyping solutions. Such innovative techniques enable precise control over electric fields without conventional microelectrode fabrication, as demonstrated in recent work by Zhao et al. [50]. To create the non-uniform electric field for DEP separation, Zhao et al. inserted the copper pad into the predesigned microfluidic channel, avoiding cleanroom-based fabrication of microelectrodes, as shown in Figure 3A. The microfluidic chip is designed with embedded copper microelectrodes within asymmetric orifices positioned on opposite sidewalls of the main channel. These asymmetric orifices create non-uniform electric fields for inducing DEP forces. The chip includes multiple inlets and outlets for precise cell routing, allowing yeast cells to migrate based on dielectric differences. The use of copper electrodes and microfabrication techniques ensures high conductivity and stable operation, critical for continuous cell sorting applications. In our group’s previous work, we developed a rapid, cost-effective xurography technique to cut PET films for multilayer construction and inkjet printing to deposit conductive ink for microelectrode arrays [51]. This fabrication avoids the need for a cleanroom and allows for quick prototyping suitable for disposable medical applications. The device features a four-layer lab-on-a-foil structure, with inkjet-printed microelectrode arrays on a flexible substrate (Figure 3B). The design includes a serpentine microchannel, cavity layers above the channel, and covers, creating a three-dimensional arrangement that is used by z-axis DEP forces. This configuration effectively separates circulating tumor cells from trapped red blood cells (RBCs) in the upper cavity layer. Similarly, Martínez-Brenes et al. developed a cost-effective method for fabricating PDMS-based DEP devices by utilizing soft lithography on copper-clad PCB templates with a laser printer (Figure 3C) [52]. This approach employs a chemically etched copper master to create micrometer-sized constrictions within a PDMS microchannel, generating electrodeless DEP. The device integrates multiple electrokinetic forces, including DEP, electroosmotic flowto trap and preconcentrate bacterial cells near the constrictions. Although these non-cleanroom methods offer cost-effective and rapid prototyping advantages, they often suffer from lower electrode resolution, potential variability in deposition, and reduced long-term stability. These limitations can impact device reproducibility and scalability, posing challenges for integration with more complex microfluidic systems.

### 2.2. Contactless Electrodes

Unlike conventional electrodes, conductive contactless electrodes are isolated within channels that do not share inlets or outlets with the primary microfluidic channel, preventing contamination and enhancing device reliability. Below, we summarize contactless conductive electrodes and their bio-applications in microfluidic devices.

Conductive liquids, known for their excellent conductivity, are increasingly used as contactless electrodes in electric microfluidic devices. By leveraging the high-resolution capabilities of soft lithography, microchannels embedded with conductive liquids enable precise electric field generation. Alinezhadbalalami et al. developed contactless liquid dielectrophoresis (cDEP), a method that uses contactless electrodes to manipulate cells via electric fields without direct contact. The device integrates electrode channels separated from the main microfluidic channel by a thin (~13 μm) PDMS membrane. The electrodes channel is filled with a conductive fluid (10× PBS), which generates a nonuniform electric field through the membrane, ensuring the integrity of both cells and electrodes. This configuration leverages soft lithography to create contactless electrode, including posts embedded in the main channel, which amplify the local field gradients near the posts for precise cell trapping. This device demonstrates the ability to enrich glioblastoma stem cells (GSCs) from heterogeneous populations based on their dielectric properties [53]. Another application utilized electrodes made from Field’s metal, a low-melting-point alloy that is introduced in a liquid state at 65 °C and solidified at room temperature within channels adjoining the main microfluidic channel [54]. The microfluidic chip is designed with orifices on one side to generate high-field regions and PDMS posts on the opposite side, creating a continuous low-field metal layer. The device leverages high-frequency pDEP (1 MHz) to selectively enrich live chemoresistant pancreatic cancer cells from a mixture of dead, apoptotic, and necrotic cells in suspension. The device demonstrates the enrichment of live chemoresistant circulating-like pancreatic cancer cells derived from drug-treated adherent cultures. Starting with a low percentage of live cells (~3%), the system enriches the live subpopulation to ~44% within 20 min while rejecting over 90% of dead cells. While contactless conductive electrodes prevent contamination and simplified electrode fabrication, they also face challenges in complexity, electric field consistency, and long-term stability. At the same time, the reliance on thin membranes or liquid electrodes may introduce variability in performance, limiting their scalability for high-throughput applications.

Droplet-based microfluidic devices have been developed to address challenges in microbial cultivation and high-precision sorting. Ho et al. designed a droplet-based microfluidic device using a T-junction for droplet generation to construct a microbioreactor with droplet trap and oscillation functions [55]. In this device, electrodes are created using a liquid indium alloy to enable the application of an alternating current (AC) electric field. This material choice supports the generation of the necessary electric field strength for droplet oscillation while maintaining compatibility with the PDMS microfluidic structure. This microbioreactor is used for microbial cultivation, specifically demonstrated with *E. coli* as a model organism, and provides a high-throughput and scalable alternative to traditional shake flask cultures. Frenzel et al. utilized a novel microfluidic platform designed for high-precision droplet sorting [56]. Fabricated using soft lithography, the device integrates patterned electrode pairs alongside channel walls, enabling precise DEP control over droplet trajectories. Custom wiring and high-voltage switching allow each electrode pair to be independently activated, facilitating robust two-way and multi-way sorting without the need for cleanroom-based fabrication. This low-cost and adaptable approach enables stable operation even under fluctuating flow rates. Using DEP forces, the device directs droplets into specific channels based on fluorescence intensity or biomarker concentration, addressing a critical need in high-throughput biological screening.

### 2.3. 3D Electrodes

Three-dimensional (3D) microstructures can effectively function as insulators when electric fields are applied to opposite sides of the structure. These insulators interact with the applied electric field to generate non-uniform electric fields around each 3D microstructure. This nonuniformity induces dielectrophoresis (DEP) phenomena, which allow for precise manipulation of particles or cells within the system. By leveraging the geometric properties of the microstructures, this approach simplifies the overall design process, eliminating the need for complex fabrication techniques traditionally required for electrode patterning. Instead, the insulator itself serves as the source of the electric field gradients, providing a cost-effective and scalable solution for various bio-microsystem applications, including cell sorting, particle trapping, and biological analysis. Additionally, insulator-based electrode arrays significantly enhance device throughput by allowing the simultaneous manipulation of multiple particles or cells. This capability is particularly advantageous for high-throughput biological assays and applications requiring parallel processing, such as rare cell isolation, drug screening, and large-scale diagnostics.

#### 2.3.1. Materials of 3D Electrodes

**Figure 4:**
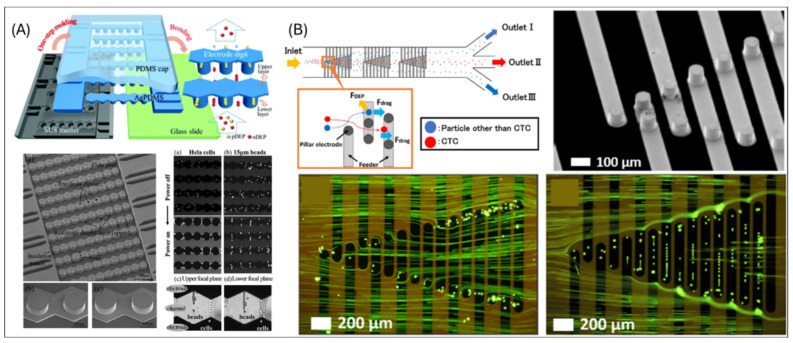
(**A**) SEM images of the Ag-PDMS device in a bottom-up position and micrographs demonstrate the vertical position discrepancies of HeLa cells and polystyrene beads under DEP activation [57]. (**B**) Illustration of mechanism of Ni-based pillar and demonstration of particle manipulation [59].

Silver-PDMS (AgPDMS) composites offer a scalable and efficient solution for integrating electrode structures into microfluidic devices. Zhang et al. demonstrated a one-step molding process to fabricate a monolithic device that integrates both fluidic channels and electrode structures using a silver-PDMS (AgPDMS) composite (Figure 4A) [57]. In this work, an AgPDMS cap layer is applied and bonded to a glass slide via oxygen plasma treatment. This process eliminates the need for additional sacrificial lithography layers and enables the seamless integration of the 3D electrode structure with the fluidic channels. The device leverages its two-layer 3D electrode design to generate a spatially non-uniform electric field for DEP cell separation. It achieves a capture efficiency of 95.4% and purity of 85.6% for live cells at flow rates as high as 0.3 mL/h. Hyler et al. also investigated a novel ultralow conductivity buffer within the similar configuration, demonstrating enhanced DEP performance by further minimizing joule heating and optimizing electric field gradients for precise cell manipulation [58].

The nickel metal (Ni) is also featured for microcylindrical (pillar) electrodes fabricated on a polyimide substrate using photolithography and electroforming. Miyamukai et al. formed a chevron-shaped array with cylindrical structures (Figure 4B) [59]. Ni electrodes are deposited into etched patterns to form Ni cylindrical electrodes, which are subsequently embedded into a microfluidic channel using adhesive tape as spacers. The Ni cylindrical electrodes generate localized non-uniform electric fields for negative DEP manipulation. When an AC electric field is applied, the design effectively repels particles or cells from the electrodes, enabling their lateral displacement. The system is optimized for separating Jurkat cells from polystyrene beads with similar sizes. With collection efficiency exceeding 95% and minimal contamination, the device demonstrates strong potential for isolating rare circulating tumor cells (CTCs) in liquid biopsies.

Carbon is also a suitable candidate to be doped in PDMS as the microelectrode. For example, a microfluidic device features 3D carbon electrode fabricated using a two-step photolithography process of SU-8 followed by pyrolysis at 1000 °C in nitrogen. The final device includes 3161 carbon electrodes, each 100 µm high and 50 µm in diameter, arranged in 218 intercalated rows. The device demonstrates high-throughput separation and enrichment of live U937 monocytes, achieving over 90% removal efficiency for dead cells under optimized flow conditions [60].

Three-dimensional silicon microelectrodes with segmented sidewall designs have been utilized in microfluidic devices to create non-uniform electric fields for DEP and electrothermal fluid rolls (ETF) [61]. These microelectrodes are fabricated using a single photomasking process that combines deep reactive ion etching (DRIE) and isotropic silicon etching, enabling precise and scalable production. The system integrates sheath flows to focus extracellular vesicles (EVs) and employs counter-rotating ETF rolls to generate helical particle trajectories. Large EVs experience strong nDEP forces near microelectrode edges and are guided to side branches, while smaller EVs follow ETF rolls and exit through the center branch. The device achieves >80% purity and recovery of small EVs like exosomes from blood serum and cell culture media.

#### 2.3.2. Configuration of 3D Electrodes

Three-dimensional electrodes can be modified into various shapes to enhance the functionality of microfluidic devices. Through the combination of the microcylindrical shape in DLD with planar platinum electrodes in a microfluidic channel, the DC and AC electric fields enhance particle separation of carboxylate microspheres and plain polystyrene beads (100 nm to 3 μm) [62]. Using standard photolithography and soft lithography techniques, a microfluidic device was designed with two sections of oval-shaped insulating posts embedded within the microchannel [63]. The gap sizes of these posts were specifically tailored to enable size-based separation of exosomes. This device employs insulator-based dielectrophoresis (iDEP) to generate localized non-uniform electric fields for exosome manipulation. Similarly, the DLD chip features triangular microposts with a side length of 55 µm, a 40 µm gap, and a tilt angle of 2.9° [64]. The DEP chip integrates 3D Ag-PDMS electrodes designed with sharp tips to create a strong electric field gradient. In the first stage, the DLD chip removes the majority of RBCs and platelets by leveraging size-based separation. Larger particles, such as CTCs, are displaced laterally, while smaller particles follow the original streamline. In the second stage, the DEP chip separates H322 lung cancer cells from WBCs based on their dielectric properties. The 3D Ag-PDMS electrodes create localized electric field gradients, enabling selective manipulation of cells via positive and negative DEP forces. The device demonstrates the ability to separate H322 lung cancer cells from diluted whole blood with a 91% separation efficiency and 80.7% purity.

Multilayer microfluidic devices integrated with 3D electrode configurations provide enhanced dielectrophoretic performance for efficient cell isolation and analysis. A replaceable porous PDMS membrane, fabricated through soft lithography, is used for constructing the microfluidic device with a micropillars array [65]. SU-8 micropillars are used as a mold to define the pores within the membrane. The membrane consists of micropores with diameters ranging from 20 to 30 µm, tailored for trapping CTCs based on size and dielectric properties. Gold interdigitated electrodes, patterned on a glass substrate using photolithography and RF sputtering, integrate with the membrane to enable DEP-based manipulation. This enables size-based separation and PC3 prostate cancer cell trapping for subsequent biological analyses.

A two-layer electrode fabrication process integrates planar bottom-layer electrodes with precisely aligned vertical sidewall electrodes, enabling 3D electric field gradients for improved dielectrophoretic performance. The planar and sidewall electrodes are fabricated using photolithography, metal deposition, and lift-off techniques. The sidewall electrodes are formed on etched vertical surfaces, which are integrated with a microfluidic device through plasma bonding. The vertical alignment of sidewall electrodes minimizes field distortion and allows manipulation in both lateral and vertical planes, which effectively reduces the clogging issue. This approach has been successfully applied to high-throughput cell sorting of CTCs from blood, achieving flow rates up to 2 mL/h [66]. Using a multilayer structure, a three-dimensional dielectrophoresis microfluidic chip incorporates serrated interdigital electrodes for CTC isolation, fabricated using wet etching and laser ablation technologies on indium tin oxide (ITO) conductive glass [67]. pDEP forces concentrate CTCs at the electrode surface, while leukocytes are suspended and flow out of the channel under negative DEP forces. The device achieves up to 94.3% isolation efficiency for hepatoma cells (SMMC-7721) at a flow rate of 1.1 mL/h. The alternative electrode fabrication, including the rapid fabrication method, contactless electrodes, and 3D electrodes, are included in Table 1 with their configuration and application and a key matrix of performance.

### 2.4. Traditional Fabrication for 2D Electrodes

This section mainly focuses on electrodes that are relatively thinner than both the microfluidic channel and the channel in open-channel microfluidic chips. These electrodes directly generate electric fields, unlike the insulator-based field generation discussed in the 3D electrodes section. The 2D electrodes can be further divided into two main types: Sidewallelectrodes and bottom-up electrodes. Sidewall electrodes generate electric fields across the channel by being positioned on opposite walls of the microfluidic channel. In contrast, bottom-up electrodes are placed beneath the primary microfluidic channel, creating an electric field from the bottom to the top of the channel. This section also highlights the common fabrication processes for 2D electrodes, which typically rely on techniques including metal deposition, photolithography, and etching. Additionally, it provides insights into the fabrication details and their connection to specific electrode design configurations.

#### 2.4.1. SidewallElectrodes Configuration

A DEP-based microfluidic device with 16 parallel sidewall ITO electrodes was fabricated using photolithography and reactive ion etching, topped with a PDMS microfluidic pool [68]. The transparent design enables real-time imaging and precise single-cell control via a closed-loop visual feedback system. By dynamically adjusting electric fields, T-lymphocytes are guided along predefined trajectories (e.g., sinusoidal or sawtooth) with submicron precision at speeds up to 50 μm/s. Lin et al. developed a microfluidic chip with ITO interdigitated electrodes fabricated via photolithography and modified with a self-assembled monolayer (SAM) of gold nanoparticles (AuNPs) [69]. Thiolate aptamers were bonded to the SAM surface through gold-sulfur interactions, enabling specific binding to A549 lung cancer cells. This chip consists of three inlets for precise sheath flow alignment and uses pDEP to direct cells to high electric field regions. The aptamer-functionalized surface significantly improved capture efficiency, retaining over 80% of A549 cells even after the electric field was removed. Similarly, Hughes et al. embedded ITO electrodes in a PDMS channel using photolithography and etching [70]. These electrodes generate AC-DEP forces to characterize the resting membrane potential (RMP) of biological cells. The platform enables label-free, non-contact cell manipulation and measures DEP responses to estimate RMP values based on Clausius–Mossotti (CM) factor variations across frequencies. This method provides accurate RMP measurements, validated against patch clamp data for different cell types, including primary suspension cells, cultured suspension cells, primary adherent cells, and cancer cell lines.

Recent developments in DEP-based microfluidic systems with bipolar electrodes (BPEs) have been applied for precise isolation of cells. For example, Chen et al. developed a wireless BPEs system using photolithography and electrode integration techniques [71]. The microfluidic chip aligns BPEs with nanoliter-scale chambers for single-cell isolation. At an optimized electric field frequency (50 kHz), the device selectively captures circulating melanoma cells (CMCs) while minimizing contamination from peripheral blood mononuclear cells (PBMCs). This system is used to isolate and analyze melanoma cells from both spike-in samples and patient blood. Similarly, right-angle BPEs integrated into a PDMS chip have been used to separate non-spherical flagellate algae [72]. Fabricated via photolithography and integrated into a PDMS chip, the electrodes generate uniform electric fields for precise manipulation of spindle- and ellipsoid-shaped cells. Numerical simulations and experiments confirm electro-orientation and dielectrophoretic assembly, achieving up to 92.78% purity for marine Euglena and 92.06% for freshwater species.

Thick electrodes are robust, vertically aligned structures used in sidewall DEP configurations, capable of generating strong and consistent electric fields. Unlike planar electrodes, which are flat and thin, thick electrodes are particularly advantageous for bio-applications requiring precise 3D manipulation. Kim et al. developed a conductive spiral channel by laminating an Ag-PDMS mixture onto an SU-8 mold, followed by bonding it to a glass substrate [73]. This design integrates hydrodynamic forces from the spiral geometry with DEP forces generated by AC voltage applied across the conductive channel walls, achieving precise exosome separation with minimal deformation. Results indicate high separation efficiency (83%) and purity for exosomes derived from lung adenocarcinoma cell media. Another device integrates thermally oxidized deterministic lateral displacement (DLD) arrays with DEP functionality to achieve high-purity exosome separation [74]. The electrode is fabricated using micrometer-scale lithography, etching, and thermal oxidation to create tapered DLD structures, enabling differential separation thresholds between layers. DEP integration employs droplet-shaped nano-pillars to separate particles based on dielectric properties and size. This approach demonstrates over 90% exosome purity from biological fluids, highlighting its potential for molecular diagnostics and biomarker isolation.

In addition to DLD structures, insulators can also be integrated into sidewall electrode configurations to create non-uniform electric fields. For example, a novel iDEP platform employs insulating porous paper fibers to generate electric field gradients [75]. This platform is fabricated by laser-cutting microchannels into fiberglass paper substrates, integrating copper foil electrodes, and encapsulating the system with PDMS sheets through thermal bonding. This scalable, cleanroom-free process takes less than 10 min. Electric fields applied across the porous structures create localized gradients, enabling DEP-based trapping of particles such as polystyrene beads and *E. coli* bacteria.

#### 2.4.2. Bottom-Up Electrodes

In this section, we focus on electrodes positioned beneath the microfluidic channel to generate electric fields from the bottom to the top, enabling applications such as cell sorting, single-cell analysis, and droplet manipulation. Specifically, we discuss the optoelectronic dielectrophoretic (ODEP) approach, traditional photolithography techniques, and the integration of bottom-up electrodes with various channel configurations.

The optoelectronic dielectrophoretic (ODEP) approach uses projected light patterns on a photoconductive surface to generate localized electric fields, enabling the movement and sorting of dielectric particles by size, shape, or dielectric properties. This method eliminates the need for physical microelectrodes, offering reconfigurable, real-time control and simplified fabrication. Amorphous silicon (a-Si) photoconductive layers integrated with ITO substrates enable the creation of dynamic, light-patterned microelectrodes, replacing traditional fixed electrode designs. For instance, an ODEP microfluidic platform is fabricated by depositing an a-Si photoconductive layer on ITO-coated glass [76]. Microchannels are constructed from laser-cut double-sided adhesive tape (30 µm thick), ITO glass substrate, and PDMS layers. A DLP projector projects light patterns onto the photoconductive layer, creating dynamic virtual electrodes. This design eliminates traditional electrode patterning and allows real-time, programmable manipulation of EVs. Similarly, a layer of amorphous silicon (a-Si) is deposited on an ITO layer using RF sputtering to serve as a photoconductive layer [77]. The bottom electrode comprises a stack of ITO, a-Si, and a passivating silicon nitride (SiN) layer on a fused silica substrate, while the top electrode is a transparent ITO layer. Light patterns for DEP manipulation are projected using a modified DLP projector and a 40× microscope objective, achieving minimum 8 µm patterns. This setup dynamically generates non-uniform electric fields, eliminating the need for fixed electrode fabrication. The platform enables precise manipulation of microbial cells, allowing for single-cell control with programmable light patterns. Using a-Si as the photoconductive material, Soong et al. employed controlled light patterns to generate non-uniform electric fields for manipulating EVs based on size and dielectric properties [78]. This platform demonstrated high-purity sorting of EVs into three size categories: small (100–150 nm), medium (150–225 nm), and large (225–350 nm), achieving 86% sorting purity. The same setup effectively facilitates precise EV manipulation and sorting with high efficiency.

The other primary fabrication method for bottom-up electrodes involves photolithography, with slight modifications to the fabrication techniques and materials to enhance precision, scalability, and compatibility with various microfluidic applications. For example, a microfluidic device with ITO-patterned glass slides, fabricated via photolithography [79], uses AC electric fields to control protein crystallization by manipulating nucleation dynamics and local conditions. Nonuniform electric fields manipulate nucleation dynamics, including induction time, nucleation rate, and crystal location. Similarly, using a gold nanowell-based plasmonic substrate with an ITO counter-electrode integrates interference lithography and reactive ion etching to create 200 nm diameter nanowells with 500 nm periodicity. These electrodes generate AC electroosmosis and DEP forces, preconcentrating EVs near the sensing surface. This enhanced capture mechanism improves detection sensitivity by 100-fold compared to conventional methods, achieving detection limits as low as ~30 EVs. The platform detects tumor-derived EVs using functionalized gold surfaces with specific antibodies (e.g., CD63, CD24, EpCAM) in a rapid 10 min assay.

By changing the electrode architecture to a vertical nanogap electrode (VNE), an electrode-insulator-electrode stack with a 100 nm gap was developed [80]. The design incorporates a patterned gold electrode on top and a planar ITO electrode on the bottom, separated by an insulating layer. This configuration generates strong DEP forces at sub-volt operation, enabling the precise capture and spatiotemporal manipulation of nanoparticles, including lipid vesicles and amyloid-beta assemblies. Weber et al. also developed a DEP microfluidic chip with ring electrode architecture, fabricated via photolithography and metal deposition on a glass substrate [81]. The design, featuring concentric ring electrodes with alternating polarities, generates high-density, non-uniform electric fields. This configuration achieves superior bacterial capture efficiency (99%) compared to traditional dot electrode designs (79%) by enhancing electric field gradients and depletion zones.

The bottom-up electrode design also enables integration with various array configurations in microfluidic chips. For example, by enhancing electric field gradients and reducing operating voltages, the platform addresses limitations of sidewall nanogap electrodes in scalability and throughput [82]. It demonstrates size-selective trapping of nanoparticles and low-voltage manipulation of biomolecules. The bottom-up design can also be used for separating cancer exosomes using a castle-walled chromium electrode array fabricated via photolithography and metal deposition [83]. With 5 µm electrode gaps creating strong non-uniform electric fields, pDEP forces capture exosomes. Fluorescently labeled exosomes from normal (human milk-derived) and cancerous (MCF7-derived) samples were analyzed in media with adjusted conductivities (10–15 mS/m). Selective cancer exosome captures with up to 57% separation efficiency were achieved by optimizing the 5 µm electrode gap, conductivity, and frequency (1 MHz). Dual nanowells are also integrated with a four-layer structure and asymmetric interdigitated electrodes to generate dielectrophoretic forces for single-cell capture [84]. Fabricated via photolithography and plasma-reactive ion etching, the design achieves >75% single-cell pairing and >97% bead occupancy, enabling co-encapsulation of human embryonic kidney (HEK) cells and functionalized beads for transcriptome and proteome analysis. Similarly, bottom-up electrodes integrated into an open-channel microfluidic device, fabricated using gold electrodes on a glass substrate, form an open micro-electro-fluidic (OMEF) biochip optimized for capturing and stretching red blood cells [85].

Micropore systems combined with bottom-up electrodes apply DEP forces to position single CTCs into individual microwell pores, ensuring efficient capture and viability [86]. This system isolated CTCs from colorectal cancer patient blood samples, enabling mutation analysis that revealed KRAS, BRAF, and PIK3CA mutations, including tumor heterogeneities. Combining an imaging cytometer with microfluidic DEP, coplanar gold electrodes fabricated via photolithography and metal deposition onto a glass substrate generate non-uniform electric fields for real-time dielectric cell characterization [87]. High-speed imaging captures cell morphology and motion, enabling simultaneous optical and dielectric analysis. Multiphysics simulations optimized velocity and Clausius–Mossotti factors to assess cell physiological states. The system discriminates viable and non-viable CHO cells, with potential applications in bioprocessing and cell phenotyping for drug response and environmental stress studies. Additionally, DEP integrated with Raman spectroscopy (SERS) employs a bottom-up electrode design to enable precise single-cell manipulation and biochemical analysis [88]. The system features interdigitated gold electrodes with nanohole arrays (250 nm diameter, 700 nm periodicity), fabricated using electron-beam lithography and lift-off. These fabrication methods ensure precise patterning and alignment of nanostructures to optimize DEP positioning and Raman signal enhancement. DEP forces position particles like polystyrene beads and glioblastoma cells (U-87 MG) on nanoholes, achieving up to an 8.1 dB SERS signal enhancement for label-free detection of cellular and molecular properties.

### 2.5. Fabrication of Microelectrode in Electrorotation

Electro-rotation is achieved by applying non-uniform electric fields generated through asymmetrical electrode arrangements. Asymmetrically placed electrodes generate dielectrophoretic (DEP) forces, inducing torque on particles and resulting in controlled rotational motion. The rotational motion can be controlled by tuning the electric field’s frequency, amplitude, or phase. The induced rotation can serve various purposes, including mixing fluids, orienting anisotropic particles (such as cells or nanoparticles), or enhancing interaction with the electrode surface. The electrode designs and fabrication methods enable fine-scale control, making them suitable for bio-related applications such as single-cell analysis, manipulation of nanoparticles, and enhanced biochemical interactions. In this section, we highlight the advancements in electrode fabrication and their impact on bioengineering applications.

Chen and Jiang utilized a four-phase electrode array made of ITO to achieve real-time imaging and precise electro-rotation [89]. The electrodes are patterned using a fiber laser marking machine (1064 nm) to create a cross-patterned electrode array with diagonal electrode distances of 160 µm. The device combines rotating electrothermal (RET) flows and DEP for particle manipulation. The RET flows, generated by rotating electric fields and temperature gradients from infrared laser heating, enable rapid particle concentration and sorting. The device demonstrates its capability to concentrate and sort various particles, including silica, metallic particles, and yeast cells.

**Figure 5:**
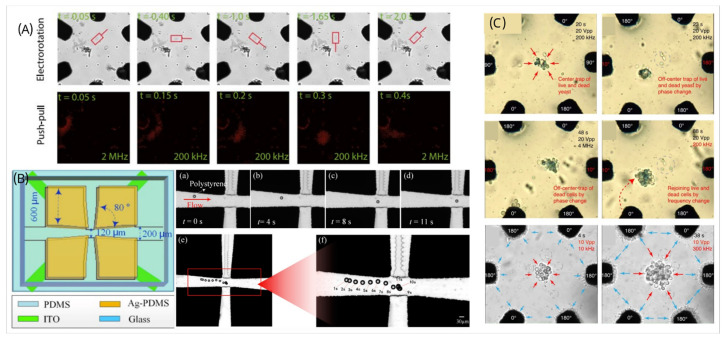
(**A**) A 30 μm polystyrene particle trapping experiment [90]. (**B**) Separation and manipulation of live and dead yeast or *B. subtilis* using frequency and phase-controlled electric fields, demonstrating selective trapping and redistribution [91]. (**C**) Bacterial cell experiments for electrorotation and push–pull effect [92].

The electrorotation-on-chip (EOC) system, fabricated with 3D carbon-PDMS (C-PDMS) electrodes (conductivity: 5 S/m) and a transparent ITO bottom electrode, incorporates a V-shaped cell trap for precise positioning [26]. The system applies AC signals to generate a rotating electric field, enabling 3D cell rotation with controlled axis and speed for precise measurements of dielectric properties like membrane capacitance and cytoplasm conductivity. This platform measures biophysical properties of HeLa, C3H10, B lymphocytes, and HepaRG cells. Like carbon-based materials, a 3D quadrupole electrode structure made of Ag-PDMS composite, integrated with ITO wiring, enables strong electric field gradients in a 38 µm microfluidic channel [90]. Fabricated by mixing silver powder and PDMS (85:15 ratio) and curing, the electrodes align precisely with the channel height for uniform force distribution. This device efficiently traps and rotates polystyrene microparticles (20–30 µm) with high precision, as shown in Figure 5A.

Julius et al. also introduced an adaptable DEP embedded platform tool (ADEPT) to achieve electrorotation; it is a portable platform with a circular microelectrode arrangement, as shown in Figure 5B [91]. The fabrication process involves photolithography and electroplating to create 10 µm thick gold electrodes on a glass substrate, enhancing electrode precision and conductivity. The platform’s six-electrode configuration, controlled via a graphical user interface (GUI), allows flexible manipulation of DEP forces. By enabling independent control of frequency, phase, and amplitude, ADEPT demonstrates efficient cell trapping, viability-based separation, and phenotype differentiation. The platform shows 94% efficiency in separating live and dead yeast and 96% efficiency in distinguishing yeast from *Bacillus subtilis*. Additionally, ADEPT facilitates cell interactions, such as phagocytosis of *E. coli* by granulocytes. There is also a low-cost microfluidic chip with a six-electrode circular array fabricated using UV lithography and electroplating on a MEMpax glass substrate, within a trapping area of 100 µm in diameter [92]. The microfluidic channels are fabricated using xurography with double-sided pressure-sensitive adhesive (PSA) tape (75 µm thick), and a laser-cut coverslip (100 µm thick) seals the structure. By applying phase-shifted AC signals, the device enables precise cell positioning, trapping, and rotation within a 3D space, demonstrating its suitability for single-cell analysis (Figure 5C).

## 3. Microelectrodes for EOF

Electroosmotic flow is a key mechanism in electrokinetic microfluidic systems, enabling precise fluid control for particle separation, biochemical analysis, and diagnostics. Fabrication of EOF-based devices involves integrating microchannels, electrodes, and surface coatings to optimize flow dynamics and performance. Planar microelectrodes, often embedded beneath the microchannels, are commonly used for generating the electric fields required for fluid manipulation. This configuration ensures uniform electric field distribution and consistent flow. Precise electrode alignment with channel geometry further enhances EOF reliability and functionality. This section reviews various fabrication methods for electroosmosis-based microfluidic device designs.

### 3.1. Photolithography Microelectrode Fraction

**Figure 6:**
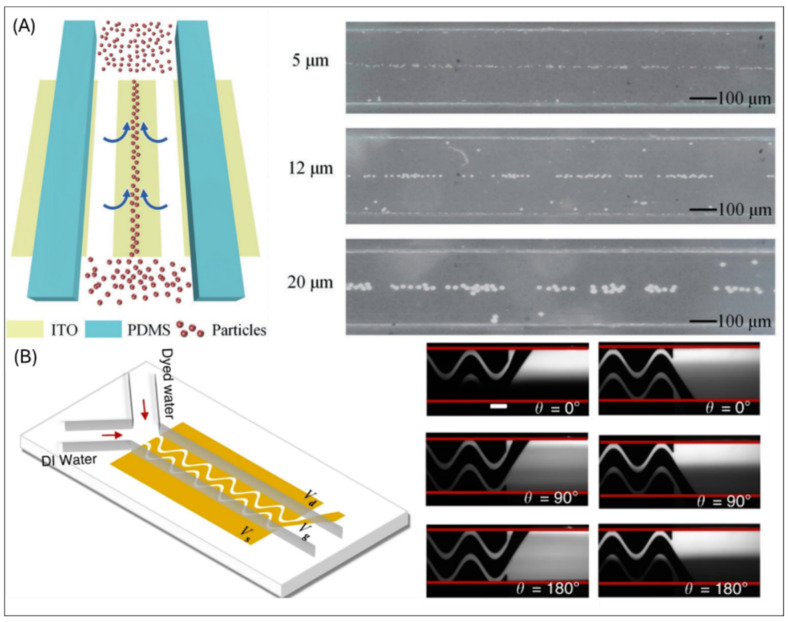
(**A**) Schematic representation of a portable visual microfluidic experimental device, along with focused illustrations of particles of varying sizes [93]. (**B**) Schematic of a phase-controlled field-effect micro-mixing device utilizing AC electroosmosis with a grayscale fluorescent image illustrating mixing performance [95].

EOF-based devices are typically fabricated using photolithography, PDMS soft lithography, and soft lithography. For instance, a portable microfluidic device integrates ITO-coated glass electrodes, fabricated via soft lithography, for multifunctional electric field regulation [93], as shown in Figure 6A. This device supports multiple electrokinetic experiments, including induced charge electroosmosis (ICEO), DEP, and thermal buoyancy convection. By applying external AC or DC electric fields, the device manipulates particle movement, focusing, and separation in microfluidic channels. Its modular design allows customization of electric field parameters by swapping top covers, ensuring adaptability. Real-time particle behavior is analyzed via a smartphone app, providing experimental feedback. Photolithography is also pivotal in developing ICEO-based platforms for enhanced protein detection [94]. A microfluidic device with open bipolar electrodes (OBPEs) is fabricated using photolithography and electrode deposition techniques. The device uses antibody-coated microcolumns placed in the ICEO flow zone to increase protein binding efficiency. By applying AC electric fields, analytes are concentrated around the OBPEs, significantly enhancing detection sensitivity.

A novel micromixer leveraging AC electroosmosis (ACEO) features three-finger sinusoidal gold electrodes fabricated via photolithography and e-beam evaporation on a borosilicate glass substrate [95], as shown in Figure 6B. A 30 µm-thick SU-8 microchannel patterned atop the electrodes is sealed with a PDMS layer. By applying phase-lagged AC voltages, the device achieves mixing efficiencies exceeding 90% at flow rates of 4 µL/min. The device enables the synthesis of lipid-based nanoparticles with phase-controlled mixing, improving particle monodispersity and concentration.

### 3.2. Alternative Microelectrode Fabrication for EOF

Beyond photolithography, several alternative fabrication methods can be employed to construct electrodes for EOF platforms. For instance, screen-printed electrodes fabricated on a polyvinyl chloride (PVC) substrate, combined with a double-sided adhesive microchannel, are applied to generate AC electroosmotic flow, enhancing the sensitivity and speed of electrochemiluminescence assays for protein and miRNA detection [96]. The AC-driven design creates high-intensity asymmetric electric fields that promote rapid antigen-antibody interactions and improve analyte capture efficiency. It successfully detects cardiac troponin I (cTnI) and miR-499-5p, the eminent biomarkers for myocardial injury diagnosis, directly from unprocessed saliva, urine, and interstitial fluids, achieving detection limits as low as 2 fg/mL and 10 aM, respectively. This fabrication method shows its potential for developing low-cost and highly sensitive point-of-care diagnostics devices.

Through integration with an electrokinetic trap, the simultaneous 2D positioning and 3D orientation are used to control nanowires, enabling manipulation with nanometer and sub-degree precision [97]. The device features a three-layer microelectrode system including ITO glass electrodes and gold microelectrodes patterned via photolithography, separated by a PDMS well. By controlling the DC and AC fields applied to this specific three-layer electrode, multiple functions can be achieved, including electrophoresis, electroosmosis, electrorotation, and electroalignment.

Cheng et al. introduced a microfluidic platform combining hybrid electrokinetic mechanisms with surface-enhanced Raman spectroscopy (SERS) for rapid pathogen identification in human blood [98]. The device integrates concentric circular electrodes, fabricated via inductively coupled plasma etching, on a roughened gold surface to enhance Raman signals. The system uses AC DEP and ACEO flows to separate and concentrate bacteria from dense blood cell mixtures within three minutes, achieving a density factor increase of approximately 1000-fold. The platform detects *S. aureus*, *E. coli*, and *P. aeruginosa* at detection limits as low as 5 × 10^3^ CFU/mL, using clear SERS fingerprints for rapid on-chip diagnostics of bacteremia and sepsis without antibody immobilization or extensive sample preparation.

Our group presented a flexible lab-on-a-foil microfluidic device designed for micromixing via AC-EOF [13]. The device fabrication employs xurography, a rapid and cost-effective technique, using biocompatible double-sided adhesive tape to define microchannels and copper films as electrodes, assembled into a disposable, low-cost platform. The device integrates staggered tooth-shaped copper electrodes, strategically positioned to generate strong AC-EOF effects. These electrodes disrupt laminar flow within the microchannels, promoting efficient mixing of fluids. Numerical simulations and experimental validations were conducted to optimize key operational parameters, including the frequency (1–10 kHz), voltage (10–20 V), and electrode geometry, achieving mixing efficiencies exceeding 90% under optimal conditions. The device demonstrates the mixing of red blood cells with aggregating agents, dextran solutions, to induce controlled aggregation.

EOF can be integrated into a novel porous microneedle (PMN) array generating transdermal electroosmotic flow to enhance drug delivery and interstitial fluid extraction [99]. The PMNs, fabricated from poly-glycidyl methacrylate (PGMA) and modified with charged hydrogels as electrodes, enable the transport of larger molecules through micropores by EOF. This research demonstrates the PMN’s capacity for delivering model drugs (e.g., dextran) and extracting glucose using pig skin samples. Additionally, it explores the potential for powering the PMN system with a built-in enzymatic biobattery.

## 4. Conclusions

This review highlights the critical role of microelectrode design and fabrication in advancing electroosmotic flow and dielectrophoretic microfluidic devices for bio-applications. The integration of advanced materials, innovative fabrication techniques, and precise electrode configurations has significantly enhanced the performance and versatility of these devices, enabling them to address challenges in diagnostics, particle manipulation, and biochemical analysis. Recent advancements in fabrication methods, such as soft lithography, photolithography, and emerging non-cleanroom techniques, have facilitated the development of cost-effective and scalable devices. Innovative approaches, including rapid prototyping, liquid contactless electrodes, and three-dimensional electrode structures, offer precise control over electric fields, improving device functionality while expanding potential applications. Materials like ITO, carbon composites, and conductive polymers have further enhanced electrode compatibility with microfluidic systems. Despite these advancements, challenges remain, including the need for more efficient and reproducible fabrication processes, minimization of Joule heating, and improved scalability for mass production. Additionally, the integration of multifunctional capabilities, such as hybrid electrokinetic forces and enhanced sensing mechanisms, requires further exploration to unlock the full potential of EOF and DEP in next-generation microfluidic devices. Future research could explore addressing these limitations by incorporating emerging fabrication technologies, such as 3D printing and nanolithography, alongside integrating novel materials and configurations with microfluidic systems to enhance device performance. Progress in these areas can further advance EOF- and DEP-based microfluidic platforms, supporting innovations in biomedical research, point-of-care diagnostics, and biochemical analysis.

## Figures and Tables

**Table 1:** Alternative electrode fabrication and bio applications.

Electrodes Type	Functionality	Application	Electrode Material	Key Matrix	Reference
Contactless liquid electrode	Cell enrichment	Glioblastoma stem cells	10× PBS		[53]
	Droplet trap and oscillation functions	Microbial cultivation for *E. coli*	Liquid indium alloy		[55]
	Cell enrichment	Pancreatic cancer cells	Field’s metal	Enrich from ~3% to ~44%	[54]
Low-cost electrode	Cell separation	Yeast cells	Copper sheet		[50]
	Cell separation	CTCs A549	Inkjet printing Ag-based conductive ink		[51]
3D electrode	Cell separation		Silver-PDMS	Capture efficiency of 95.4%	[57]
	Cell separation	Jurkat cells	Nickel metal	Efficiency exceeding 95%	[59]
	Cell enrichment	U937 monocytes	Carbon PDMS	90% removal efficiency	[60]
	Particle purification	EVs	Silicon	80% purity	[61]
DLD combination	Cell separation	H322 lung cancer cells	Ag-PDMS electrodes	80.7% purity	[64]
	Cell trapping	PC3 prostate cancer cell	SU-8 micropillars		[65]
Multilayer microfluidic devices	Cell isolation	Hepatoma cells (SMMC-7721)	Indium tin oxide (ITO) conductive glass	94.3% isolation efficiency	[67]
